# Deconstructive and Divergent Synthesis of Bioactive Natural Products

**DOI:** 10.3390/molecules28176193

**Published:** 2023-08-22

**Authors:** Si-Hua Hou, Feng-Fan Zhou, Yi-Hang Sun, Quan-Zhe Li

**Affiliations:** Shanghai Key Laboratory of Chiral Drugs and Engineering, College of Pharmaceutical Sciences, Shanghai Jiao Tong University, Shanghai 200240, China; zff_the_flash@sjtu.edu.cn (F.-F.Z.); syh031030@sjtu.edu.cn (Y.-H.S.); qzli2016@sjtu.edu.cn (Q.-Z.L.)

**Keywords:** bioactive natural products, total synthesis, deconstructive synthesis, divergent synthesis

## Abstract

Natural products play a key role in innovative drug discovery. To explore the potential application of natural products and their analogues in pharmacology, total synthesis is a key tool that provides natural product candidates and synthetic analogues for drug development and potential clinical trials. Deconstructive synthesis, namely building new, challenging structures through bond cleavage of easily accessible moieties, has emerged as a useful design principle in synthesizing bioactive natural products. Divergent synthesis, namely synthesizing many natural products from a common intermediate, can improve the efficiency of chemical synthesis and generate libraries of molecules with unprecedented structural diversity. In this review, we will firstly introduce five recent and excellent examples of deconstructive and divergent syntheses of natural products (2021–2023). Then, we will summarize our previous work on the deconstructive and divergent synthesis of natural products to demonstrate the high efficiency and simplicity of these two strategies in the field of total synthesis.

## 1. Introduction

Since the synthesis of urea in 1828, humans have opened the door to organic synthesis. With the development of isolation and identification techniques and organic synthesis methods, people are pushing the limits of complex molecular synthesis, especially complex natural products, such as vitamin B12 and anemone toxin. Natural products are the main source of innovative medicines, such as artemisinin and paclitaxel. From 1981 to 2020, over 50% of the small molecule drugs approved by the FDA were derived from natural products or the parent molecular structure of natural products [1]. It can be seen that innovative drug development based on active natural products has played a crucial role in the field of original drug research and development. However, active natural products are often very scarce in nature and have low direct druggability. Therefore, research on the total synthesis of active natural products can not only solve the problem of limited natural resources and prepare a large amount of active natural products but also enable the rapid synthesis of natural product analogues with diverse skeletons and functional groups, building a natural product-like compound library to conduct extensive biological activity screening and drug development research. It has important scientific significance and potential application value in the field of natural product synthesis and new drug development. Thus, the development of practical synthetic methods and efficient synthetic strategies in natural product total synthesis has always been an active field in organic synthesis and has attracted great attention from synthetic chemists.

**Deconstructive synthesis**, namely building new (and often more challenging) structures through bond cleavage and the formation of easily accessible moieties, has emerged as a useful design principle in preparing complex bioactive natural products and other target molecules [2]. The basic logic of deconstructive synthesis is to construct a “hardly accessible” skeleton from an “easily accessible” skeleton through skeletal reorganization. Meanwhile, the rapid construction of polycyclic molecular frameworks, the precise instillation of multi-stereocenters bearing crowed quaternary carbon centers, and the efficient induction of versatile functional groups should also be highlighted. It also facilitates the continuous modification of key advanced intermediates to improve the overall efficiency of the synthetic route. The subtlety of deconstructive synthesis often lies in its creative approach of building intricate molecular frameworks, resulting in the design of easily accessible intermediates that could significantly reduce the challenge of total synthesis. The development of deconstructive synthesis strategies is often inspired by the molecular structural characteristics of natural products, which also tests the creativity and personalized perspective of synthetic chemists.

**Figure 1:**
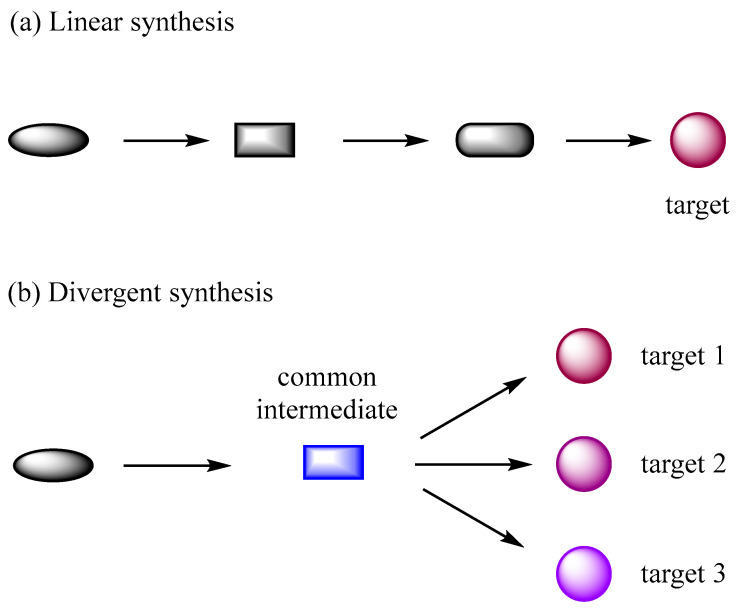
Schematic explanation of linear and divergent synthesis.

**Scheme 1:**
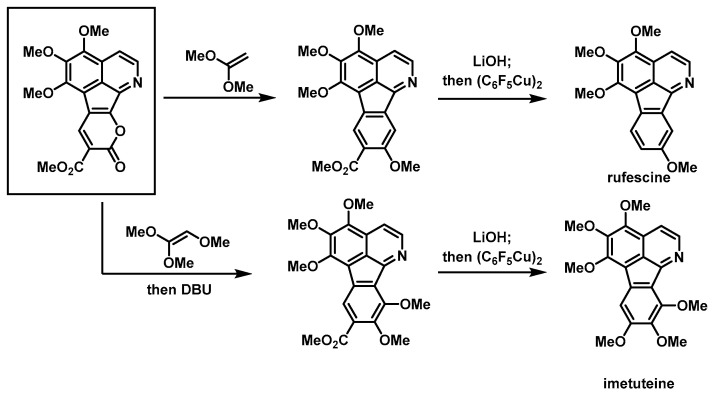
Boger’s divergent total syntheses of rufescine and imetuteine.

**Divergent synthesis**, in short, involves working from one common intermediate to many natural products (Figure 1). In general, a class of natural products often shares a similar chemical structure with various functional groups or different molecular frameworks that can be constructed from a common intermediate in biosynthesis. The original definition of divergent synthesis was proposed and demonstrated by Boger in 1984 (Scheme 1) [3]. “Divergent” is defined as a common intermediate (preferably an advanced intermediate) being converted, separately, to at least two natural products. Applications of divergent synthesis include not only preparing molecules in the same family but also accessing natural products that have the same molecular skeletons from different families. Divergent total synthesis, also defined as “collective total synthesis” by MacMillan, is when multiple skeletons of natural products are prepared from a versatile common intermediate [4].

Compared with linear synthesis that can only achieve one natural product at a time, the divergent strategy provides a more efficient approach to access many natural products with similar or different molecular skeletons from a versatile molecule through a number of routine chemical operations. In addition, the divergent synthesis of natural products is also more conducive to building a natural-product-like compound library to conduct more biological activity screening and innovative drug development based on the bioactive natural products.

The application of both strategies requires organic chemists to be familiar with the skeleton, functional group, oxidation state, and other characteristics of the target molecules. One of the most challenging steps is to design a suitable advanced intermediate that can be easily prepared and quickly converted to as many target molecules as possible. Meanwhile, the common intermediate should also be close to the target molecule to reduce the total number of synthesis steps.

During the past decade, many research groups have developed diverse, novel synthetic methodologies to realize a lot of impressive approaches for the divergent and deconstructive synthesis of natural products. Previous literature on the divergent strategy in natural product total syntheses from 2013 to June 2017 has been summarized in *Chemical Reviews* [5]. Excellent reviews focusing on deconstructive synthesis through rearrangement reactions already exist, providing more comprehensive perspectives for authors who have interests in this field [6,7,8,9,10,11,12].

In the last three years, many research groups have accomplished a lot of creative synthetic routes to syntheses of natural products with complex structures and broad biological activities. In this review, we will first introduce five excellent examples to demonstrate the synthetic utilities of the deconstructive and divergent strategies in natural product total synthesis. Then, we will summarize our previous work on the deconstructive and divergent synthesis of bioactive natural products to demonstrate the high efficiency and simplicity of these two strategies in natural product total synthesis. After a brief introduction of the respective bioactive natural products, we will discuss the details of the synthetic routes and how to combine the deconstructive and divergent strategies in target molecules.

## 2. Selected Deconstructive and Divergent Syntheses of Natural Product (2021–2023)

**Scheme 2:**
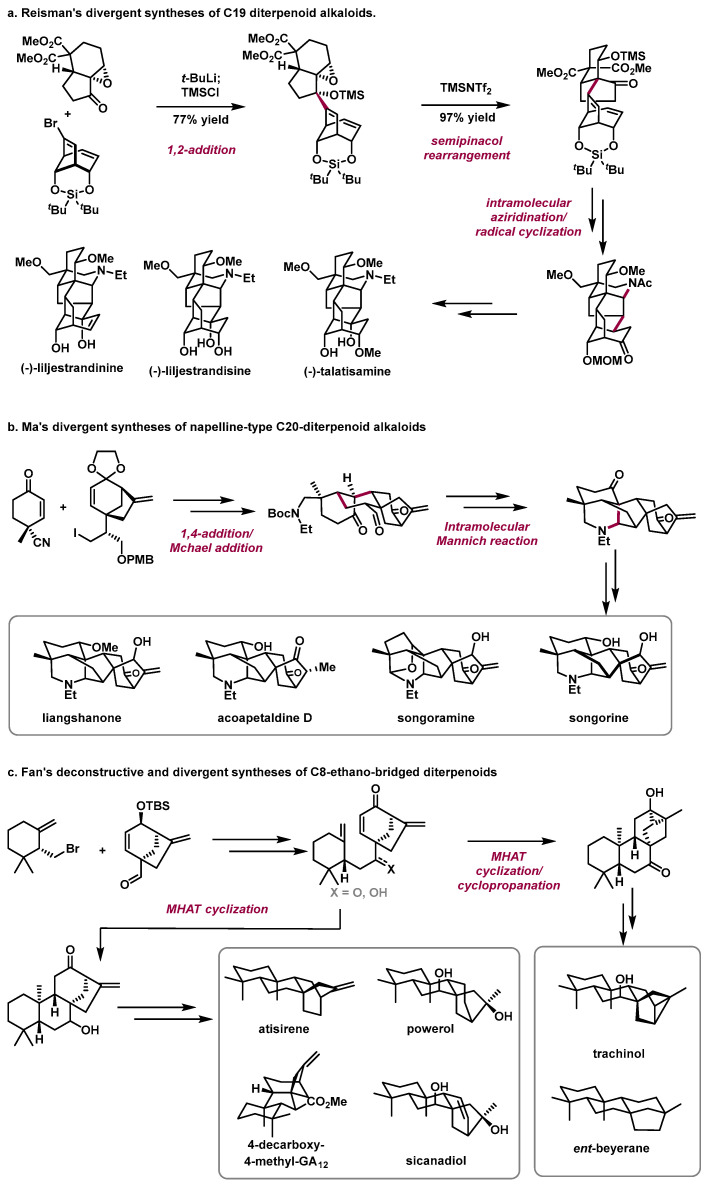

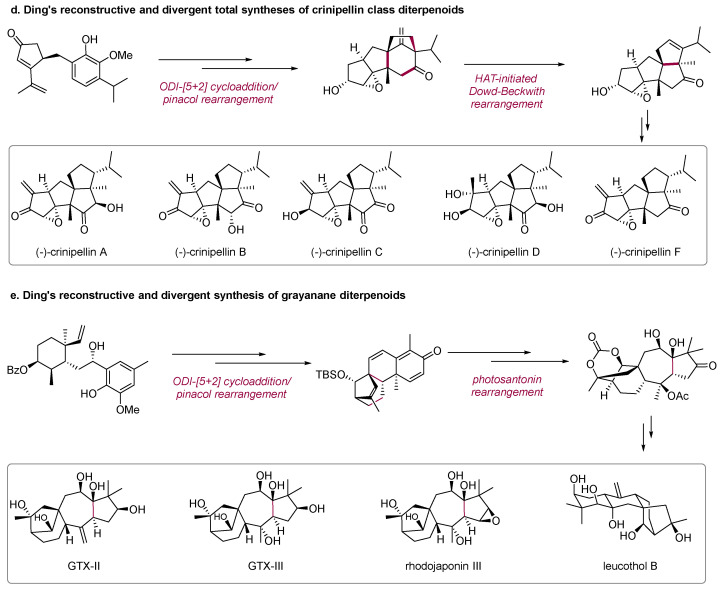
Selected examples of deconstructive and divergent syntheses of natural products.

In 2021, the Reisman group from the California Institute of Technology reported the divergent total syntheses of three C19 diterpenoid alkaloids: (−)-talatisamine, (−)-liljestrandisine, and (−)-liljestrandinine from phenol (Scheme 2a) [13]. The highlights of this work include (1) a 1,2-addition/semipinacol rearrangement sequence to efficiently couple two complex fragments and construct the quaternary carbon center and (2) an intramolecular aziridination and radical cyclization to assemble the pentacyclic skeleton of the target alkaloids.

In 2022, the Ma group from the Shanghai Institute of Organic Chemistry reported an asymmetric divergent approach to the total synthesis of six napelline-type C20-diterpenoid alkaloids in a convergent manner (Scheme 2b) [14]. The highlights of this work include (1) a diastereoselective intermolecular Cu-mediated conjugate addition to couple the two fragments; (2) an intramolecular Michael addition reaction to construct the tetracyclic skeleton; and (3) an intramolecular Mannich cyclization to rapidly construct the azabicyclo [3.2.1] octane motif of the target molecules.

In 2023, the Fan group from Lanzhou University published a deconstructive and divergent synthesis of nine C8-ethano-bridged diterpenoids based on late-stage skeletal diversification in the longest linear 8 to 11 steps starting from readily available chiral materials (Scheme 2c) [15]. Crucial advanced intermediates with different structural skeletons were rapidly constructed through regioselective and diastereoselective metal-hydride hydrogen atom transfer (MHAT) cyclization from the *multi*-reactivity of densely functionalized polycyclic substrates.

In 2022, the Ding group from Zhejiang University developed deconstructive and divergent syntheses of eight tetraquinane diterpenoids through a HAT-initiated Dowd−Beckwith rearrangement reaction for the efficient assembly of diversely functionalized polyquinane frameworks (Scheme 2d) [16].

In 2023, the Ding group finished the deconstructive and divergent syntheses of nine grayanane diterpenoids that belong to five distinct subtypes from a common advanced tetracyclic intermediate which was prepared through a tandem intramolecular oxidative dearomatization-induced (ODI) [5 + 2] cycloaddition/pinacol rearrangement to construct [3.2.1]-bicyclic skeleton and a photosantonin rearrangement to assemble the 5/7 bicyclic framework (Scheme 2e) [17].

## 3. Divergent Syntheses of Fawcettimine-Class *Lycopodium* Alkaloids

**Scheme 3:**
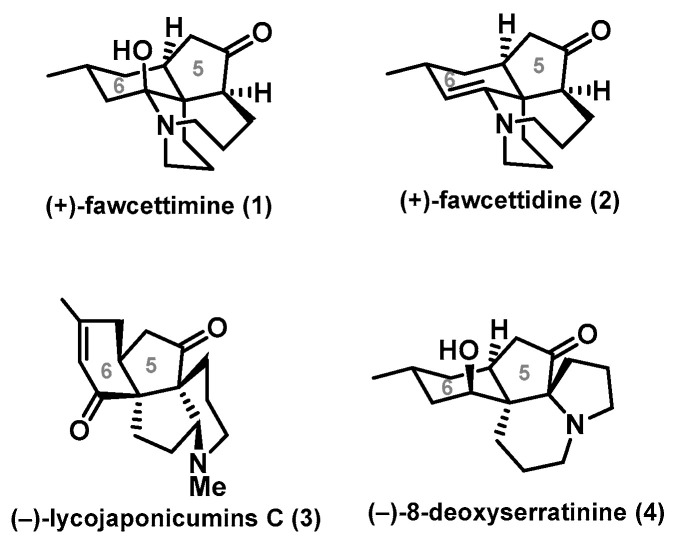
Structures of representative fawcettimine-class *Lycopodium* alkaloids.

*Lycopodium* alkaloids are structurally complex natural products with quinolizine, pyridine, or *α*-pyridone, originally identified in the *Lycopodium* genus. *Lycopodium* alkaloids exhibit important biological activities. For example, Huperzine A is a potent inhibitor of acetylcholinesterase (AChE) and shows promise in the treatment of Alzheimer’s disease (AD). The fawcettimine-class *Lycopodium* alkaloids are a class of structurally unique alkaloids with complex and unique skeletons bearing quaternary carbon centers, such as fawcettimine (**1**); fawcettidine (**2**); lycojaponicumins C (**3**); and 8-deoxyserratinine (**4**) (Scheme 3) [18]. In particular, lycojaponicumins C (**3**), isolated by Yu and co-workers in 2012 from the traditional Chinese medicine *Lycopodium japonicum*, exhibits lipopolysaccharide (LPS)-induced pro-inflammatory factors in BV2 macrophages [19]. The fawcettimine-class *Lycopodium* alkaloids feature fused tetracyclic skeletons, including two common *cis*-hydroindene (6/5 bicycle) motifs and two diverse ring systems bearing quaternary carbon centers. The *Lycopodium* alkaloids have attracted great attention from synthetic chemists and medicinal chemists for their unique chemical structures and broad biological activities. During the past decade, several elegant approaches for the total synthesis of *Lycopodium* alkaloids have been reported [20].

In 2013, Tu’s group reported a creative approach to divergent synthesis of four fawcettimine-class *Lycopodium* alkaloids, namely, fawcettimine (**1**), fawcettidine (**2**), lycojaponicumins C (**3**), and 8-deoxyserratinine (**4**) [21]. In this work, the authors designed a common intermediate which can transform to alkaloids **1**–**4** in a divergent manner. Meanwhile, a practical methodology to quickly construct the *cis*-hydroindene skeleton was designed and developed to demonstrate that natural product total synthesis can promote the development of novel synthetic methodologies and synthetic strategies.

**Scheme 4:**
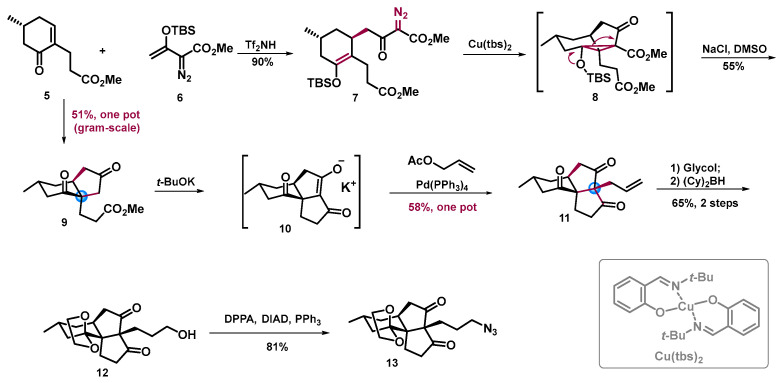
Synthesis of tetracyclic azide (**13)**.

After optimization of the Mukaiyama–Michael addition conditions, coupling of two simple building blocks **5** and **6** was realized in the presence of triflimide to afford silyl ether **7** with excellent yield and high diastereoselectivity (Scheme 4). Then, Cu(tbs)_2_-catcalyzed carbene cyclization designed by our group was successfully conducted, and decarboxylation of the resulting cyclization product provided the desired ketone **9** with a *cis*-6/5 bicyclic skeleton and a quaternary carbon center with a 55% yield [22]. The bicycle **9** could also be scalable prepared with good yield by using a one-pot operation. Notably, this novel methodology offers an efficient way to construct the *cis*-6/5 bicyclic skeleton from simple building blocks which can apply to the total synthesis of other complex natural products. Then, Dickmann condensation of the ketone ester **9** gave an enolate of tricyclic trione **10**, which in situ reacted with π-allylpalladium complex to deliver the tricyclic compound **11** with angular 6/5/5 tricyclic molecular framework bearing two quaternary carbon centers. Selective protection of the ketone on the six-membered ring and hydroboration of the terminal double bond of **11** gave the desired alcohol **12**, which could readily be converted into the common intermediate, the tricyclic azide **13,** through the Mitsunobu reaction.

**Scheme 5:**
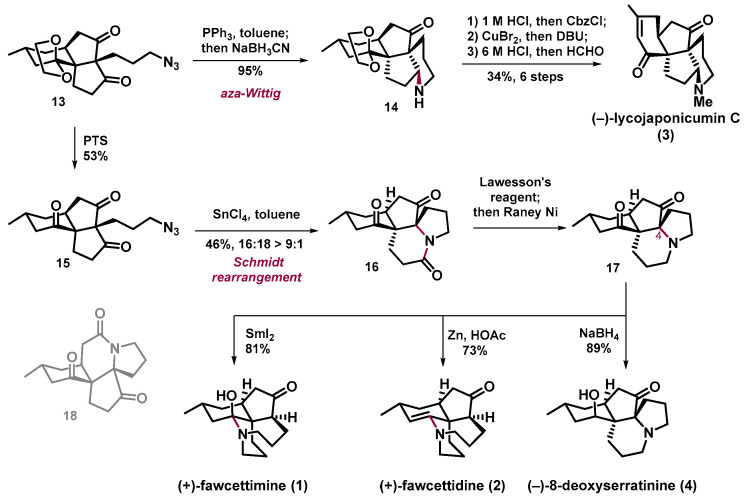
Divergent syntheses of fawcettimine-class *Lycopodium* alkaloids fawcettimine (**1**), fawcettidine (**2**), lycojaponicumins C (**3**), and 8-deoxyserratinine (**4**).

The tricyclic azide **13** possesses an angular 6/5/5 tricyclic framework, two contiguous quaternary carbon centers, two carbonyl groups, and an azide chain and can serve as a versatile common intermediate to divergent synthesis alkaloids, namely, fawcettimine (**1**), fawcettidine (**2**), lycojaponicumins C (**3**), and 8-deoxyserratinine (**4**), via the deconstructive strategy. On the one hand, the regioselective *aza*-Wittig reaction and reduction of **13** yielded tetracyclic amine **14**, which was transformed to (−)-lycojaponicumins C (**1**) through several chemical operations to install the double bond and N-methyl group of the target molecule (Scheme 5). On the other hand, the regioselective Schmidt rearrangement of **15**, obtained from deprotection of **14**, was realized and provided the desired tetracyclic lactam **16** in a useful synthetic yield. Treatment of **16** with Lawesson’s reagent followed by a reduction of the resulting sulfo-lactam with Raney Ni gave tetracyclic amine **17** with a 66% yield. Then, a late-stage skeletal diversification of tetracyclie **17** was conducted to synthesize three alkaloids with different skeletons. Thus, compound **17** could divergently transform to the following: (−)-8-deoxyserratinine (**4**) via selective NaBH_4_-reduction; (+)-fawcettimine (**1**) under an SmI_2_/THF reductive condition via selective C4-N cleavage/*aza*-ketalization; and (+)-fawcettidine (**2**) under a Zn /HOAc harsh reductive condition via C4-N cleavage/*aza*-ketalization/dehydration.

In this work, the Tu group have explored a divergent and deconstructive strategy for the total syntheses of four fawcettimine-class *Lycopodium* alkaloids, namely, fawcettimine (**1**, 10 steps), fawcettidine (**2**, 12 steps), lycojaponicumins C (**3**, 12 steps), and 8-deoxyserratinine (**4**, 12 steps) from simple building blocks **5** and **6**. A major innovation of this strategy involved the design of a versatile common intermediate **13**, which can synthesize not only the fawcettimine-class *Lycopodium* alkaloids but also other complex natural products. Other highlights of this work include (i) two consecutive one-pot procedures to rapidly assemble the angular 6/5/5 tricyclic framework bearing two contiguous quaternary carbon centers at the early stage; (ii) a highly regioselective *aza*-Wittig reaction, Schmidt rearrangement, and selective C4-N cleavage to construct the differently sized rings of target natural products for late-stage skeletal diversification.

## 4. Deconstructive Syntheses of *Cyclopiane* Class Tetracyclic Diterpenes

**Figure 2:**
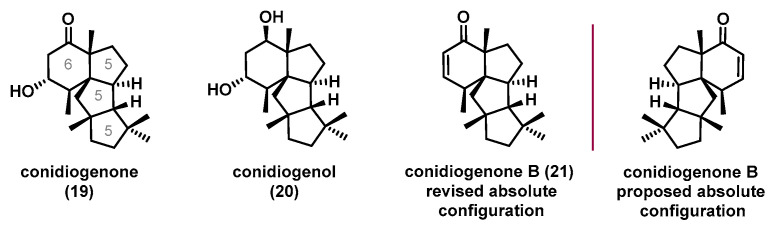
Representative bioactive *Cyclopiane*-class tetracyclic diterpenes.

The *Cyclopiane* class tetracyclic diterpenes are a class of unique bioactive natural products isolated from fermentation of marine-derived entophytic fungi of the *Penicillium* genus [23,24,25]. Conidiogenone (**19**) and conidiogenol (**20**) exhibit potent conidiation-inducing activity, while conidiogenone B (**21**) shows high antibacterial activities (Figure 2). The structures of the *Cyclopiane* class tetracyclic diterpenes feature a highly fused and strained tetracyclic (6/5/5/5) skeleton, 6–8 consecutive chiral centers, and four sterically hindered quaternary carbon centers. The *Cyclopiane*-class tetracyclic diterpenes have attracted great attention for their unprecedented chemical structures and important biological activities. During the past decade, several elegant approaches for the total syntheses of *Cyclopiane* class tetracyclic diterpenes have been reported [26,27,28,29]. The first total syntheses of three *Cyclopiane* class tetracyclic diterpenes, namely, conidiogenone (**19**), conidiogenol (**20**), and conidiogenone B (**21**), was accomplished by the Tu group in 2016 through the use of a well-designed semipinacol rearrangement as a key step to construct the requisite spirocyclic (6/5) skeleton and sterically hindered quaternary carbon center of the target molecules [26].

**Scheme 6:**
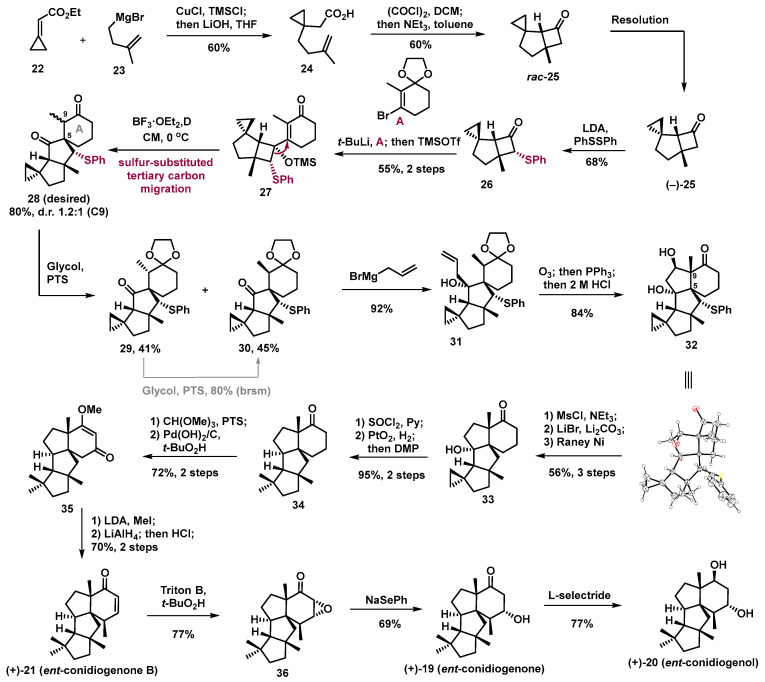
Deconstructive syntheses of the *Cyclopiane*-class tetracyclic diterpenes (**19**–**21**).

This work started with the preparation of enantioenriched cyclobutanone **25** that was obtained via chiral resolution of racemic **25**, which was prepared in two steps from simple building blocks **22** and **23** via 1, 4-addition, and [2 + 2] cyclization (Scheme 6). The well-designed vinyl cyclobutanol silylether precursor **27** was successfully synthesized by coupling known vinyl bromide **A** and phenylthiol-cyclobutanone **26**, which was obtained from enantioenriched **25** and phenyl disulphide in the presence of LDA. The phenylthioether group was firstly introduced to adjust the electron density of expected carbon in semipinacol rearrangement, aiming to enhance a dominated migration of the expected carbon of the rearrangement precursor **27**. Fortunately, under the BF_3_·OEt_2_/DCM condition, the expected semipinacol rearrangement occurred and provided the desired spirocyclic product **28** bearing the correct quaternary carbon center with an 80% yield and with a ratio of 1.2:1 at the C9 position. The structure and stereocenters of **28** were confirmed via X-ray crystallographic analysis of its derivative **32** (*vide infra*). After serval chemical operations, the last five-membered ring of the target molecule was successfully constructed via acid-promoted Aldol cyclization of aldehyde, which was obtained from terminal olefin **31** through Ozonization. After removal of the unnecessary functional group on the triquinane (5/5/5 tricyclic) motif, tetracycle **35** was obtained with a high yield. Then, stereoselective installation of a methyl group in the presence of LDA and LiAlH_4_ reduction of the resulting ketone, followed by quenching with HCl aqueous solution to install the enone motif, provided conidiogenone B (**21**) with a 70% yield. However, the positive optical rotation and CD spectrum data of synthetic conidiogenone B were opposite to those of the natural occurring one. Thus, the correct absolute configuration of naturally occurring conidiogenone B (**21**) is, in fact, the enantiomer of the originally assigned absolute configuration. Meanwhile, stereoselective epoxidation of conidiogenone B, followed by reduction of the resulting epoxide with NaSePh, afforded conidiogenone (**19**) with a 54% yield over two steps. Finally, diastereoselective reduction of conidiogenone (**19**) with L-selectride gave another diterpene conidiogenol (**20**) with a 77% yield.

In this work, the Tu group achieved the first total synthesis of the *cyclopiane* class tetracyclic diterpene conidiogenone B and its transformation into conidiogenone and conidiogenol by installing a SPh “directing” group on the expected migratory carbon of the precursor to perform regio- and diastereo-selective semipinacol-type rearrangement. The absolute configuration of naturally occurring conidiogenone B (**21**) is also corrected through this synthesis. More importantly, the 6/5/5 tricyclic ring system of the target molecule was rapidly constructed via well-designed semipinacol rearrangement in one step, which not only constructed a crowded ring system bearing a quaternary carbon center but also reserved versatile functional groups for the introduction of the last ring and vicinal quaternary carbon center (C9), making the synthetic route more pretrial and efficient.

## 5. Asymmetric Total Syntheses of Serrulatane and Amphilectane Diterpenoids

**Figure 3:**
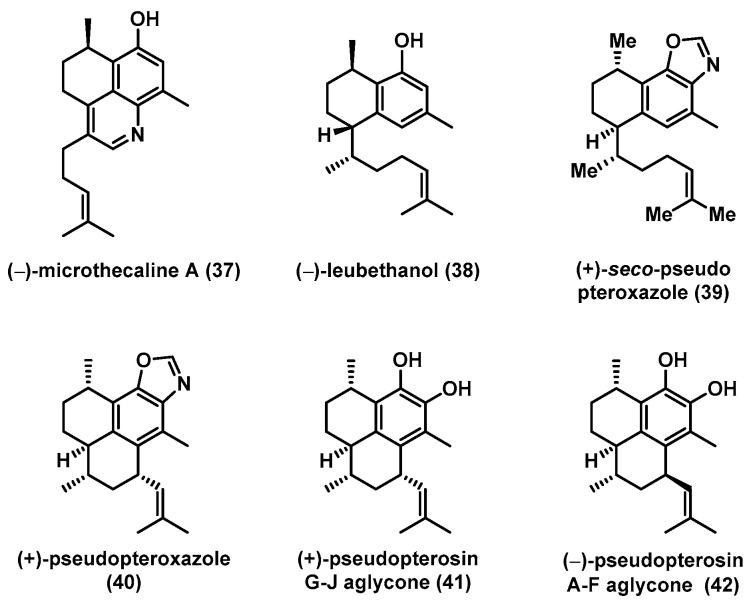
Representative of bioactive serrulatane and amphilectane diterpenoids.

Serrulatane and amphilectane diterpenoids are a class of polycyclic natural products that exhibit broad biological activities, such as *anti*-malarial, *anti*-tuberculosis, and *anti*-bacterial properties. (Figure 3). (−)-Microthecaline A (**37**) is a unique nitrogen-containing serrulatane-type diterpenoid that exhibits *anti*-malarial activity against *Plasmodium falciparum* (IC_50_ = 7.7 μM) [30]. (−)-Leubethanol (**38**) is a serrulatane diterpene isolated from the roots of *Leucophyllum frutescens* with important *anti*-tuberculosis activity (MIC 6.25–12.50 μg/mL) [31]. Because of their wide-ranging biological activities and challenging structures, synthesis of serrulatane and amphilectane diterpenoids have attracted great attention from synthetic chemists, and several total syntheses have been accomplished in the past decade. In 2020, the Dong group developed a divergent and innovative approach to quickly access diverse serrulatane diterpenoids, namely, (−)-microthecaline A (**37**), (−)-leubethanol (**38**), (+)-*seco*-pseudopteroxazole (**39**), amphilectane diterpenoids (+)-pseudopteroxazole (**40**), (+)-pseudopterosin G–J aglycone (**41**), and (−)-pseudopterosin A–F aglycone (**42**), by using a Rhodium catalytic C–C/C–H cascade reaction to construct the common skeleton, α-tetralone [32].

**Scheme 7:**
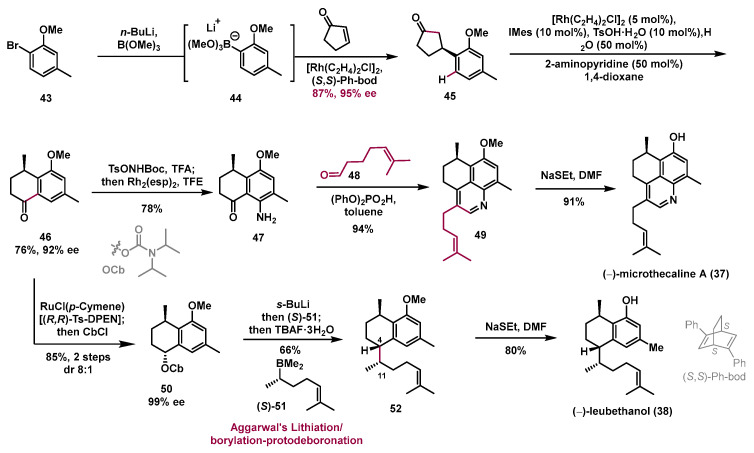
Total synthesis of (−)-microthecaline A (**37**) and (−)-leubethanol (**38**).

In this work, asymmetric Rhodium-catalyzed 1,4-addition was applied to the preparation of the enantioenriched 3-arylcyclopentanone **45**, which was the precursor of the C–C activation reaction (Scheme 7). Compound **45** could be conveniently accessed with a 87% yield with 95% ee through asymmetric 1,4-addition of 2-cyclopentenone with fresh prepared arylborate salt **44**, which was obtained from commercially available 1-bromo-2-methoxy-4-methylbenzene **43** and B(OMe)_3_ in the presence of *n*-BuLi. Under the previously reported C–C/C–H activation conditions, the desired α-tetralone **46** was obtained with a 76% yield and 92% ee. Aniline **47** was isolated with a 79% yield from **46** through Kurti/Falck’s Rhodium-catalyzed amination. The quinoline **49** was prepared via Friedländer condensation of aniline **47** and aldehyde **48** in the presence of (PhO)_2_PO_2_H with an excellent yield. Finally, removal of the methyl group by NaSEt provided (−)-microthecaline A (**37**) with a 91% yield. Meanwhile, α-tetralone **46** could also convert into carbamate **50** through Noyori asymmetric reduction and carbamoylation of the resulting alcohol. Notably, under Aggarwal’s lithiation-borylation reaction condition, compound **52** was obtained with a 66% yield by coupling the freshly prepared chiral organoborane **51** with carbamate **50**. Finally, removal of the methyl group with NaSEt gave (−)-leubethanol (**38**) with an 80% yield.

**Scheme 8:**
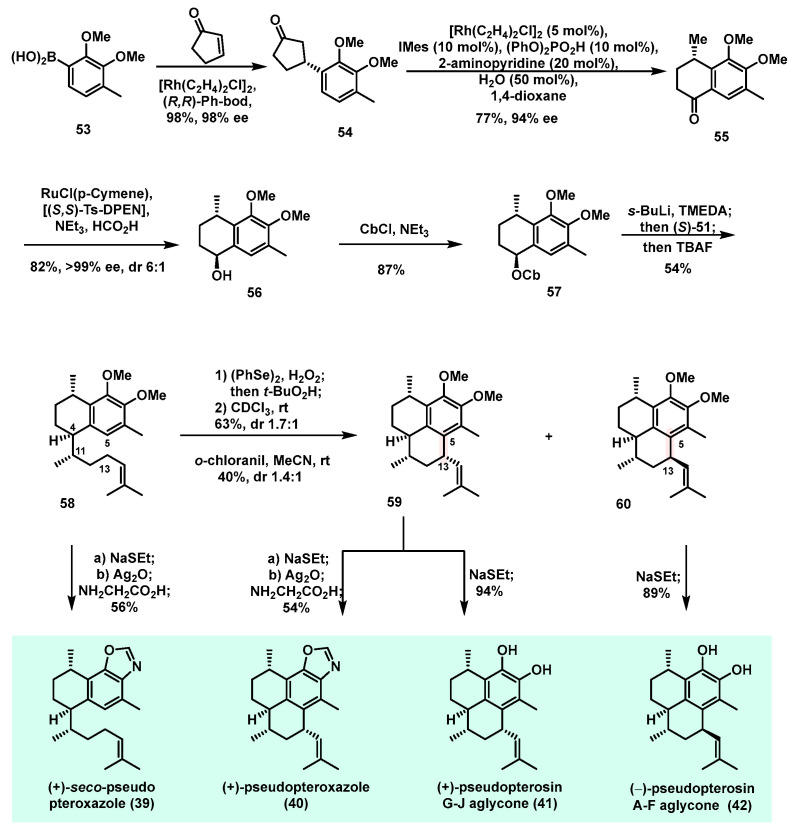
Total syntheses of terpenoids (+)-*seco*-pseudopteroxazole (**39**), (+)-pseudopteroxazole (**40**), (+)-pseudopterosin G–J aglycone (**41**), and (−)-pseudopterosin A–F aglycone (**42**).

Using the synthetic route of preparation of compound **52**, the common advanced intermediate **58** was obtained from aryl boronic acid **53**, 2-cyclopentenone, and chiral organoborane **51** in five steps with a 30% overall yield (Scheme 8). Then, a direct cross-dehydrogenative-coupling (CDC) of **58** was realized under *o*-chloranil/MeCN conditions to afford the desired tricyclic cyclization products **59** and **60** with a 40% combined yield with a ratio of 1.4:1. Finally, (+)-*seco*-pseudopteroxazole (**39**) and (+)-pseudopteroxazole (**40**) were synthesized from **58** and **59** via deprotection and one-pot oxidative oxazole formation, respectively. Pseudopterosin G–J aglycone (**41**) and (−)-pseudopterosin A–F aglycone (**42**) were prepared from **59** and **60** through deprotection, respectively.

In this work, the use of catalytic C–C/C–H activation of 3-arylcyclopentanones as a key step has been illustrated in the enantioselective total synthesis of a range of diterpenoids, namely, (−)-microthecaline A (**37**, five steps), (−)-leubethanol (**38**, six steps), (+)-*seco*-pseudopteroxazole (**39**, seven steps), (+)-pseudopteroxazole (**40**, eight steps), (+)-pseudopterosin G–J aglycone (**41**, eight steps), and (−)-pseudopterosin A–F aglycone (**42**, eight steps). This strategy can accelerate asymmetric construction of the poly-substituted tetrahydronaphthalene cores, therefore significantly simplifying the overall synthesis. This is a nice example of the design of common advanced intermediates for divergent synthesis of two classes of bioactive natural products through a deconstruction strategy. With the power of the new synthetic methodology and strategy, the synthetic approach to these diterpenoids is significantly shorter than that in previous work, which could remarkably accelerate the investigation of their potential as drug candidates in drug discovery and development.

## 6. Deconstructive Synthesis of Morphine Alkaloid (−)-Thebainone A

**Figure 4:**
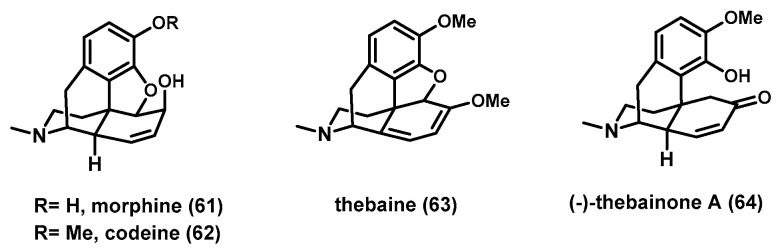
Morphine and related alkaloids.

Morphine and codeine have attracted great attention for their powerful biological activity and medical applications (Figure 4). Modification of the morphine alkaloids is still an active field in drug discovery. Therefore, developing a new asymmetric synthetic route of morphine alkaloids and their analogues is highly desirable for exploring their potential utilities in drug discovery and development. In 2021, the Dong group reported a concise enantioselective deconstructive synthesis of the morphine alkaloid thebainone A for the first time, as well as formal synthesis of codeine and morphine from commercially available materials [2,33]. The high efficiency of the synthetic strategy is enabled by an asymmetric Rh-catalyzed C–C activation reaction (cut-and-sew) to access the all-carbon fused-rings structure and quaternary carbon center.

**Scheme 9:**
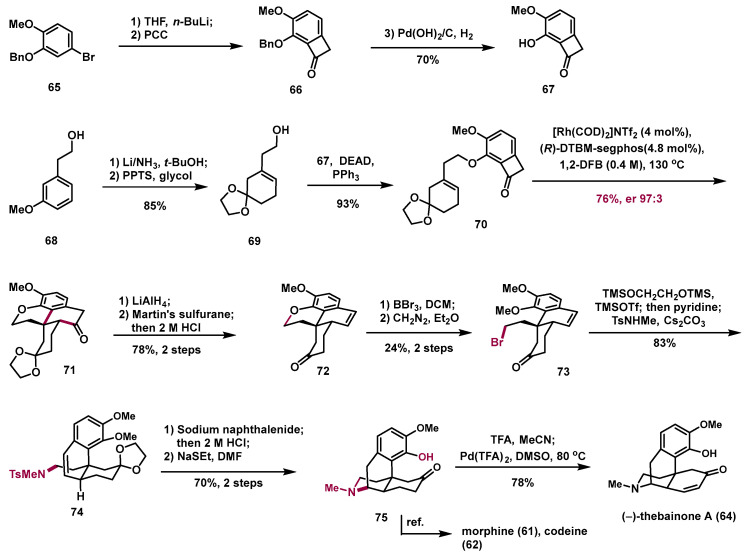
Deconstructive synthesis of morphine alkaloids (−)-thebainone A (**67**).

According to the previously synthetic route, benzocyclobutenone **67** was prepared from commercially available compound **65** in three steps on a decagram scale with a 70% overall yield, while alcohol **69** was accessed through Birch reduction and ketal formation of commercially available anisole **68** (Scheme 9). Mitsunobu coupling of phenol **67** and alcohol **69** delivered the desired precursor **70** with a 93% yield. Because of the acid-sensitive ketal, sterically hindered trisubstituted olefin, and relatively long linker of compound **70**, the efficiency of C–C activation is a challenge. After screening a series of C–C activation reaction conditions, we successfully realized Rh-catalyzed enantioselective C–C activation and obtained the desired tetracyclic compound **71** with a 76% yield with 97:3 er on a gram scale. Notably, compound **71** contains the all-carbon fused 6/6/6 rings bearing the quaternary carbon centers of the target molecules. This step not only sets the requested stereochemistry at the C13 and C14 positions but also forms all the C−C bonds present in the natural products. Compound **71** transformed into styrene **72** with a 78% yield via LAH-reduction, elimination, and deprotection. Cleavage of the C−O bond with BBr_3_, followed by methylation of the resulting diphenol provided the desired alkyl bromide **73**. Then, a one-pot sequence of ketal installation and SN2 amination of alkyl bromide smoothly delivered sulfonamide **74**, which could provide the common intermediate **75** through formal hydroamination under a sodium naphthalenide condition to construct the piperidine and selectively deprotect the more sterically hindered methyl ether in the presence of NaSEt. The common intermediate **75** could not only transform into morphine alkaloid (−)-thebainone A (**64**) through desaturation under Stahl’s condition but also serve as a known precursor for the syntheses of codeine (**61**) and morphine (**62**) [13].

In this work, the Dong group developed a novel strategy for the synthesis of the morphine alkaloid (−)-thebainone A (**67**). Key steps included (i) construction of the all-carbon 6/6/6 tricyclic skeleton bearing a quaternary carbon center through an asymmetric Rh-catalyzed C–C bond activation reaction from easily accessible benzocyclobutenone; (ii) construction of a piperidine ring from dihydropyran through C–O bond cleavage with BBr_3_ and C-N bond formation in the presence of sodium naphthalenide. This creative approach is an excellent example of the application of the deconstructive strategy in the total synthesis of natural products. Furthermore, a reoptimized catalytic C–C bond activation condition was also discovered, with good substrate scope and potential application in the synthesis of other polycyclic natural products.

## 7. Conclusions and Future perspective

Divergent and deconstructive synthesis are becoming common and important strategies in the total synthesis of natural products. How to effectively combine the two strategies has attracted the attention of synthetic chemists, which often results in the two strategies playing a role of “1 + 1 > 2”, such as bringing greater convenience in the innovative total synthesis of natural products.

The synthesis of complex natural products has always been a challenging objective in the Organic Chemistry field. It is exciting to see that more and more complex products, such as complex alkaloids and terpenoids, are being synthesized in the laboratory. Efficient and simple synthesis can provide reliable access to natural products thus facilitating innovative drug discovery and development. We hope that this review will attract more synthetic chemists to pay more attention to deconstructive and divergent synthesis, and to design elegant approaches to synthesize more natural products with complex polycyclic structures and broad biological activities.

## Data Availability

Not applicable.
